# Preliminary evaluation of a mindfulness intervention program in women with long COVID dysautonomia symptoms

**DOI:** 10.1016/j.bbih.2025.100963

**Published:** 2025-02-11

**Authors:** Elizabeth Vandenbogaart, Matthew Figueroa, Diana Winston, Steve Cole, Julienne Bower, Jeffrey J. Hsu

**Affiliations:** a Division of Cardiology, Department of Medicine, David Geffen School of Medicine at UCLA, Los Angeles, CA, USA; b UCLA Mindful at UCLA Health, University of California, Los Angeles, Los Angeles, CA, USA; c Department of Psychiatry & Biobehavioral Sciences, David Geffen School of Medicine at UCLA, Los Angeles, CA, USA; d Department of Psychology, University of California, Los Angeles, Los Angeles, CA, USA; e Division of Cardiology, Department of Medicine, Veteran Affairs Greater Los Angeles Healthcare System, Los Angeles, CA, USA

## Abstract

**Background:**

The symptom burden for patients with Long COVID-associated dysautonomia is high, yet there are currently no effective treatments. Mindfulness programs reduce psychological and physical symptoms as well as inflammatory gene expression in a variety of medical conditions. The study aim was to evaluate the effect of a six-week mindfulness program in women with Long COVID dysautonomia symptoms.

**Methods:**

Using a single arm, pre- and posttest design, women aged 18–54 years with Long COVID and orthostatic intolerance suggestive of dysautonomia were recruited from a single center. Participants attended a standardized, six-week, virtual mindfulness program. An active stand test and 6-min walk test (6MWT) were performed at baseline and post-intervention. Self-reported measures of physical and mental health symptoms collected at baseline, post-intervention and 4 week follow up included the composite autonomic symptom score (COMPASS-31), perceived stress (PSS), anxiety (GAD7), depression (PHQ8), COVID-19 event specific distress (IES-R), fatigue (FSI), sleep (ISI), well-being (MHC-SF), resilience (CD-RISC 10), and quality of life (SF-20). The effects on conserved transcriptional response to adversity (CTRA) were examined by next-generation sequencing of dried whole blood samples.

**Results:**

Twenty participants were enrolled with a mean age of 39.9 years (range 21–52 years). No significant changes were observed for the active stand test or 6MWT. A significant reduction in insomnia severity (ISI: 16.6 vs. 13.6; p = 0.001) was observed post-intervention, but scores reverted toward baseline levels at 4-week follow-up. No significant improvements were seen in autonomic symptoms, anxiety, perceived stress, depression, well-being, or COVID-19 related distress. Pro-inflammatory CTRA gene expression decreased significantly from pre-to post-intervention (*p* = 0.004). Declines in CTRA gene expression were most significant among those with 3 COVID-19 positive events (*p* = 0.01), followed by 2 events (p = 0.04) and 1 event (p = 0.05). Declines in CTRA gene expression did not vary significantly as a function of recent illness, COVID-19 hospitalization, demographic characteristics, or general medical history.

**Conclusion:**

A virtual, six-week mindfulness program may improve sleep quality in women with Long COVID dysautonomia. While no objective improvement in dysautonomia symptoms were observed, our findings suggest a favorable effect of the mindfulness intervention on inflammatory and antiviral biology with a decrease in CTRA gene expression. Nonetheless, the symptom burden in this population is very high, and more attention is needed to provide effective multi-modal clinical therapies to this population.

## Highlights

• High symptom burden identified in women with Long COVID orthostatic intolerance.
• Mindful Awareness Practices (MAPs) program was associated with a significant reduction in self-reported insomnia severity.
• Mindfulness intervention was associated with a significant decrease in pro-inflammatory gene expression.
• MAPs may improve psychological well-being for women with Long COVID.

## 1 Introduction

While there are varied definitions for post-acute sequelae of COVID-19 (PASC), commonly referred to as Long COVID, the Centers for Disease Control and Prevention (CDC) estimates the prevalence of Long COVID among non-hospitalized individuals after COVID-19 as ranging from 7.5 to 41% (Ford et al., 2023). The total burden of Long COVID on society is profound, with 2–4 million people in the United States estimated to be out of work due to Long COVID, resulting in up to $168 billion in lost earnings (Bach, 2022).

Patients with Long COVID commonly describe a myriad of persistent debilitating symptoms that can include fatigue, post-exertional malaise, and “brain fog” or difficulties with concentration and memory (Klein et al., 2023; Michelen et al., 2021; Nalbandian et al., 2021; Thaweethai et al., 2023). A particularly menacing constellation of symptoms for Long COVID patients is related to dysautonomia caused by dysfunction of the autonomic nervous system (Barizien et al., 2021). Dysautonomia in Long COVID predominantly affects young to middle-aged women causing disabling symptoms of fatigue, labile blood pressure, orthostatic hypotension, palpitations, and dyspnea resulting in significant exercise intolerance (Becker, 2021; Bisaccia et al., 2021; Davis et al., 2023). Dysautonomia has been estimated to occur in up to 30–67% of patients with Long COVID (Fedorowski and Sutton, 2023; Ladlow et al., 2022), with 2–14% developing postural orthostatic tachycardia syndrome 6–8 months after infection (Ormiston et al., 2022).

While the physiological symptom burden of Long COVID is high, the psychological impact is also substantial. A meta-analysis of 31 studies of COVID-19 survivors reported the pooled prevalence of depression was 45%, anxiety 47%, and sleeping disturbances 34% (Deng et al., 2021). The psychological effects of Long COVID-related symptoms on patients may be the result of multiple factors that, when compounded, increase vulnerability. These may include the immune response to the virus itself (including a resultant decrease in serum serotonin levels), disabling physiologic symptom burden, stressors associated with the uncertain time course of sequelae, loss of independence, and economic implications (Si et al., 2021; Wong et al., 2023). Although the underlying pathophysiological mechanisms of Long COVID are not fully known, one of the prominent theories is a chronic inflammatory state after the initial COVID-19 illness (Tandon et al., 2024). Serum proteomic analyses of people with Long COVID have identified the presence of protein signatures indicative of persistent inflammation, particularly proteins associated with Type II interferon and NF-kB signaling, in a subset of patients (Talla et al., 2023) Thus, there is reason to believe that interventions that target these inflammatory pathways may improve symptoms in this syndrome.

While hundreds of clinical trials for Long COVID are ongoing (Ramonfaur et al., 2024), including the NIH-supported RECOVER studies (Horwitz et al., 2023), treatment approaches for Long COVID thus far have primarily been reliant upon expert consensus opinion, including treatments adapted from those for syndromes with similar clinical profiles (e.g., myalgic encephalomyelitis/chronic fatigue syndrome [ME/CFS]), focusing on targeted symptom management (Blitshteyn et al., 2022; Griffin, 2024). Examples of currently recommended approaches include the use of antidepressants and slow-paced breathing techniques (Griffin, 2024). Such therapies are recommended to address symptoms of anxiety, depression, and decreased vagal tone.

Navigating the significant Long COVID symptom burden profiles can present substantial challenges to patient physical and psychological well-being and quality of life (Nalbandian et al., 2021), highlighting the importance of a comprehensive, holistic patient management approach focusing on improving physical, mental, and social wellbeing. Use of complementary, evidence-based approaches that are innovative and may be more easily available than traditional options are important considerations. Mindfulness practice may be one such option.

Over the past decade, many studies have demonstrated the efficacy of mindfulness-based interventions (MBI) for a variety of chronic conditions (Zhang et al., 2021). Mindfulness has been defined as a practice that involves the purposeful, nonjudgmental focus and awareness on the present moment experience through varying techniques of intentional paced breathing, meditation, or attention focused on body sensations, emotions and thoughts (Kabat-Zinn, 2013). The theoretical premise of mindfulness practice is that an individual's response to stressors or unpleasant experiences becomes reflective and not reactive. Mindfulness practice can disrupt negative thoughts, emotions or sensations and increase one's own capacity to regulate a state of acceptance leading to positive psychological outcomes (Hofmann and Gómez, 2017; Kabat-Zinn et al., 1992). MBI studies have shown benefit in reduction of stress, anxiety, depression (Antonova et al., 2021; Blanck et al., 2018), and improvement in fatigue and sleep quality (Bower et al., 2021). MBIs have also been related to inflammatory processes (Ballesio et al., 2023; Black and Slavich, 2016; Bower and Irwin, 2016; Jang et al., 2017). Our group has shown that a six-week mindfulness intervention - Mindful Awareness Practices - is associated with decreases in pro-inflammatory gene expression in breast cancer survivors (Bower et al., 2015, 2023; Boyle et al., 2019). MBIs were significantly associated with post-treatment increase in anti-inflammatory cytokines (Ballesio et al., 2023).

MBIs have been shown to be effective in mitigating symptoms that are similar to those in patients suffering with Long COVID, but studies involving mindfulness interventions in the Long COVID patient population have been limited to date. A four-week neuro-meditation program was found to reduce cognitive impairment and improve physical and mental fatigue, muscle and joint pain, symptoms of depression and anxiety, mood disturbances, and sleep quality in patients with Long COVID (Hausswirth et al., 2023). These participants exhibited symptom profiles that overlap with those experienced by persons with dysautonomia (e.g., physical and mental fatigue, poor sleep, anxiety), and thus this study suggests the potential benefit of MBI in Long COVID-related dysautonomia. Additional work assessing the effectiveness of an amygdala and insula retraining program combined with mindfulness training to improve the quality of life in patients with long COVID is currently underway (Gasión et al., 2023). Currently, while two prior studies have evaluated the effects of slow-paced breathing on heart rate variability (Da Silva et al., 2023; Mauro et al., 2024), there are no known studies specifically addressing the effects of MBIs on Long COVID symptom profiles related to dysautonomia.

In the present work, we performed a pilot study to evaluate the feasibility and effects of an evidence-based mindfulness intervention on the physical and psychological health, quality of life, and inflammatory gene expression in women with Long COVID-related dysautonomia symptoms.

## 2 Materials and methods

### 2.1 Study design

The aim of this single arm, prospective, pilot study was to assess the feasibility, acceptability, and preliminary efficacy of a group MBI on women with Long COVID and objective evidence of dysautonomia using a one group pretest/posttest design at UCLA Health, a single, academic medical center in Los Angeles, CA. The UCLA Institutional Review Board approved the study procedures (UCLA IRB #22–001020), with adherence to the ethical standards of the IRB and Health Insurance Portability and Accountability Act-compliant compliance protocols. There were no ethical concerns related to the interventions or potential conflicts of interest. Clinical trial registration was completed prior to enrollment (ClinicalTrials.gov NCT05566379). Informed consent was obtained from all participants.

### 2.2 Study participants

From December 20, 2022–April 27, 2023, a convenience sample of participants were recruited from the UCLA Long COVID Program, Clinical Trials.gov, and internet-based Long COVID support network referrals. Inclusion criteria included: 1) women age 18–54 years, 2) previous COVID-19 infection confirmed by PCR testing and diagnoses of Long COVID, and 3) dysautonomia confirmed by objective testing (e.g., autonomic reflex screen, active stand test), with 4) the ability to comprehend English and complete assessments and patient-reported surveys, and 5) availability of a smartphone, tablet, or computer with Internet access. Exclusion criteria were: 1) inability to participate in the virtual intervention or complete outcomes surveys, and 2) current participation in regular mindfulness practice, and/or 3) current enrollment in another COVID-19 related study. Informational flyers were provided in the UCLA Long COVID clinics and electronically by request. Interested participants completed screening to determine eligibility in the clinic or by telephone. Due to requirements of the funding organization, this pilot study only included women 18–54 years old.

Participants completed an in-person baseline visit, a six-week group mindfulness intervention over a virtual platform, an in-person post intervention visit, and a 4-week follow up questionnaire. In-person visits included physical testing, self-reported questionnaires and blood sampling before and 1–2 weeks after the intervention was completed. Pre-specified primary outcomes were changes in hemodynamic parameters on orthostatic testing, exercise tolerance as assessed by 6-min walk distance, and health-related quality of life. Secondary outcomes were changes in inflammatory gene expression as assessed by conserved transcriptional response to adversity (CTRA) on blood sampling, states of perceived stress, anxiety, depressive symptoms, event (specifically, COVID-19) related distress, fatigue, sleep, given their prevalence in Long COVID as well as assessment of well-being, and resilience. A follow up questionnaire package and patient experience survey was administered electronically 1 month post intervention to complete the study.

### 2.3 Intervention

Participants completed a group-based, six-week mindfulness intervention, Mindful Awareness Practices (MAPs), developed by the Mindful Awareness Research Center (MARC) at UCLA. In response to COVID-19 pandemic precautions, participants met virtually for six weekly, 2-h group sessions. If a participant was unable to attend a class session, a prerecorded make-up session was provided.

MAPs is a standardized intervention that provides theoretical context and materials on mindfulness, relaxation, the mind-body connection, and the various mindfulness meditation practice techniques (Black et al., 2015; Bower et al., 2021; Boyle et al., 2019). The MAPs program was delivered through lecture and a group discussion process led by an experienced mindfulness instructor who received specialized training at the UCLA MARC. MAPs class concepts include solving obstacles to effective practice, working with difficult thoughts and emotions, managing pain, and cultivating positive emotions. Written materials were provided with a summary of information covered each week. Between the weekly MAPs class sessions, participants were instructed to perform daily self-practice mindfulness exercises (5–20 min) at home and integrate the informal use of mindfulness in daily life. Participants were asked to keep a self-reported log of their daily practice. Intervention adherence was monitored by attendance taken at each group session.

### 2.4 Data acquisition

Baseline demographic data including ethnicity, gender, age, highest level of education, marital status, comorbidities (e.g., hypertension, obesity, diabetes), COVID-19 disease severity, number of severe acute respiratory syndrome-related coronavirus 2 (SARS-CoV-2) infections, COVID-19 vaccination status, and previous experience with mindfulness practice were collected from the self-reported survey and the electronic medical record (EMR).

### 2.5 Physical measure outcomes

The active stand test and 6-min walk test (6MWT) were used to evaluate physical outcome measures of dysautonomia symptoms and functional capacity. For the active stand test, heart rate, blood pressure, and symptoms were assessed after resting lying down, then immediately upon standing and after 2, 5 and 10 min (Finucane et al., 2019). For the 6MWT, the distance an individual was able to walk over a total of 6 min was recorded. The individual was allowed to self-pace and rest as needed as they walk back and forth along a flat marked walkway. Baseline heart rate and oxygen saturation were measured at the start and end of the 6 min. The patient's baseline and post-test perceived dyspnea and fatigue were rated using the Borg scale.

### 2.6 Inflammatory gene expression

Effects on inflammatory activity focusing on pro-inflammatory gene expression and associated transcription factors were assessed. Aspects of the immune system gene expression were examined from whole dried blood samples collected using the Tasso-M20 device (Tasso, Inc.; Seattle WA). The device was applied to the upper arm of each participant for approximately 5 min to collect four 20 μl blood samples at pre- and post-intervention. RNA was extracted from the dried blood samples (Qiagen RNeasy), converted to cDNA using a highly efficient mRNA-targeted reverse transcription system (Lexogen QuantSeq 3′ FWD), and sequenced on an Illumina NovaSeq instrument in the UCLA Neuroscience Genomics Core Laboratory, all following the manufacturer's standard protocols for low-mass RNA samples. Sequencing targeted 10 million 100-nt sequencing reads per sample (achieved mean = 10.2 million), each of which was mapped to the GRCh38 reference human transcriptome using the STAR aligner (average 91.9% mapping rate), with gene-specific transcript abundance values normalized to read counts per million total mapped reads and log2-transformed for analysis by linear statistical models relating gene expression levels to time point (pre-vs post-intervention; repeated measure). The primary analysis tested for intra-individual change over time (i.e., unadjusted, with no covariates involved). To evaluate the robustness of primary analysis results, a series of exploratory secondary analyses was conducted that each controlled for a single additional covariate (as multiple covariate control was not feasible in this small sample). Covariates controlled for in these individual secondary analyses included demographic characteristics (e.g., age, ethnicity, education, marital status, employment status, income), pre-existing conditions (e.g., depression, anxiety, PTSD, smoking, hyperlipidemia, diabetes, hypertension, mindfulness practice), and psychometric measures. Secondary analyses also controlled for changes in leukocyte subset abundance over time (using mRNA markers of CD4^+^ and CD8^+^ T cells, B cells, NK cells, and monocytes; CD3D, CD4, CD8A, CD19, CD16/FCGR3A, CD56/NCAM1, and CD14). Additional exploratory secondary analyses tested whether pre-to post-intervention change varied with the number of COVID-19 positive events from diagnosis to study entry (Events × Time interaction), number of COVID-19 hospitalizations (Hospitalizations x Time), or recency of infection (Recent x Time). Inflammatory gene expression was assessed using a standard set of 3 alternative bioinformatic approaches as previously described (Cole et al., 2020), including: 1) analysis of a pre-specified set of stress-related gene transcripts (measuring the Conserved Transcriptional Response to Adversity; CTRA (Cole, 2019)) that contrasts average expression of 19 pro-inflammatory gene transcripts with 34 Type I interferon related transcripts (with the latter sign-inverted to reflect their inverse contribution to the CTRA), 2) TELiS promoter-based bioinformatic analysis of NF-kB (inflammatory) and IRF (interferon) transcription factor activity in genes showing >1.5-fold change over time (Cole et al., 2005, 2020), and 3) Transcript Origin Analyses (TOA) testing whether the same set of differentially expressed genes derived disproportionately from classical and/or nonclassical monocytes (which are the key cellular mediators of inflammatory gene expression in circulating immune cells) (Cole et al., 2011, 2020).

### 2.7 Outcomes measures questionnaires

Self-reported measures of physical and psychological outcomes were collected at baseline, post intervention and at 4-week follow-up. Valid and reliable instruments for autonomic symptom severity (Composite Autonomic Symptom Scale 31, COMPASS-31), perceived stress (Perceived Stress Scale, PSS) (Cohen et al., 1983), anxiety (Generalized Anxiety Disorder, GAD-7) (Spitzer et al., 2006), depression (Patient Health Questionnaire, PHQ-8) (Kroenke et al., 2009), COVID-related distress (Impact of Event Scale – Revised, IES-R) (Weiss and Marmar, 1996), fatigue (Fatigue Symptom Inventory, FSI) (Hann et al., 1998), sleep quality (Insomnia Severity Index, ISI) (Bastien, 2001), resilience (Connor-Davidson Resilience Scale, CD-RISC) (Campbell-Sills and Stein, 2007), and quality of life (20-Item Short Form Health Survey, SF-20) (Stewart et al., 1988) were used.

### 2.8 Evaluation of the patient experience

Post intervention, three questions regarding the application and barriers to practice were collected through focused patient interviews with recorded responses. The three open-ended interview questions were: 1) In what ways did you find mindfulness helpful? 2) In what ways did you find it difficult? 3) What do you think we can do in future to improve this mindfulness intervention experience?

At 4-week follow-up post intervention, participants were asked to complete a mindfulness patient experience survey containing three 5-point Likert scale statements: (1) I found the mindfulness intervention helpful, (2) I liked the mindfulness intervention virtual format, and (3) I will likely continue to practice mindfulness.

### 2.9 Data analysis

Given the exploratory nature of this proof-of-concept pilot study, and the short timeline of data collection, a sample size of 20–30 participants was estimated. Statistical analysis included descriptive statistics, paired *t*-test, analysis of variance (repeated measures ANOVA), and Fisher's exact test, where appropriate. For analysis of the pre-specified set of CTRA indicator genes, analyses utilized mixed effect linear models treating each gene as a repeated measurement on each individual and testing for change in average indicator gene expression from pre-to post-intervention (Time main effect) while specifying a random individual-specific intercept to control for correlated residuals within individuals and across time points. These analyses were conducted using SAS PROC MIXED with maximum likelihood estimation. For analyses of differential gene expression, all transcripts showing >1.5-fold change over time from pre-to post-intervention served as input into TELiS and TOA analyses, with statistical testing based on bootstrap resampling of linear model residual vectors (which controls for within-individual correlation in gene expression). The feasibility of the MBI was evaluated through descriptive statistics of frequency of intervention attendance record, number of participants and number of those who completed all surveys.

Patient acceptability of the intervention was measured through tabulation of three 5-point Likert scale statements regarding benefit, format of intervention and likelihood of continued mindfulness practice. Patient feedback regarding the mindfulness experience was analyzed from the 3-question focused interview responses. Narration on overall impressions, benefits, challenges, and suggested improvements were uploaded into an Excel file and coded within each category. Similar codes were clustered, and themes extracted.

## 3 Results

### 3.1 Participant characteristics

Fifty-four women were screened for eligibility and 20 women were enrolled (Fig. 1). The cohort was divided into 2 sequential interventional groups: the first group with 7 and the second group with 13 participants. All 20 participants completed baseline physical testing, self-reported questionnaires, and blood sampling. Sixteen participants (80%) completed the post-intervention and 4-week follow-up assessments. Self and family member health and medical demands were reported as the barriers for the four participants who were unable to complete the post intervention study visit. Women ranged in age from 21 to 52 years (mean 39.9 years). 40% were white, 50% were married or with a domestic partner, 60% had completed college, and 45% were employed full-time. Comorbid medical and psychological conditions of the group included hypertension (10%), hyperlipidemia (20%), diabetes (5%), smoking history (5%), pre-existing anxiety (40%) and pre-existing depression (30%). The number of SARS-CoV-2 infection exposures prior to study entry revealed 65% of the participants had 1 positive SARS-CoV-2 infection, 25% had 2 positive SARS-CoV-2 infections and 10 % had 3 positive SARS-CoV-2 infections. In relation to severity of COVID-19 infection 10% experienced a COVID-19 related hospitalization. Baseline demographic and disease related variables are reported in Table 1.

**Fig. 1:**
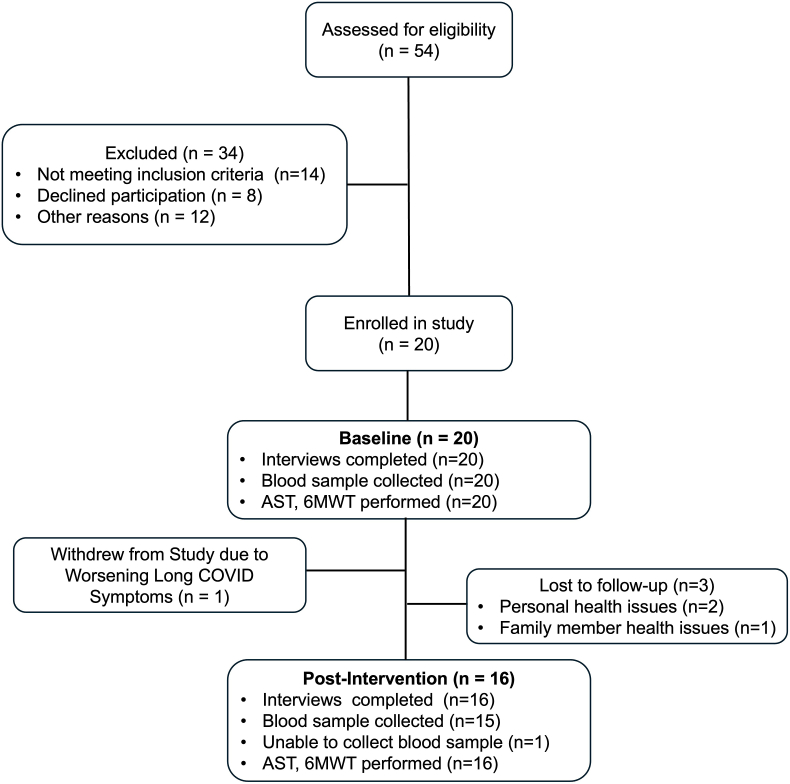
Clinical Flow Diagram. Abbreviations: AST, active stand test; 6MWT, 6-min walk test.

**Table 1:** Baseline sociodemographic and medical characteristics of study participants.

	Total (*N* = 20)
Age, mean (range)	39.9 years (21–52)
	
Race/Ethnicity, N (%)	
Black/African American	2 (10%)
Hispanic/Latino	4 (20%)
White/Caucasian	8 (40%)
Other	5 (25%)
Education, N (%)	
Less than College	1 (5%)
College Degree	7 (35%)
University/Post Graduate Degree	12 (60%)
Marital Status, N (%)	
Married/Domestic Partnership	10 (50%)
Divorced/Separated	5 (25%)
Never Married/Single	5 (25%)
Employment Status, N (%)	
Employed Full-Time	9 (45%)
Unemployed	1 (5%)
Disability	6 (30%)
Homemaker	3 (15%)
Student Full-Time	1 (5%)
Household Income, N (%)	
<$50,000	3 (15.79%)
$50,001-$75,000	4 (21.05%)
$75,001-$100,000	4 (21.05%)
>$100,000	8 (42.11%)
History of COVID-19 Hospitalization, N (%)	2 (10%)
Positive COVID Tests Prior to Study Entry, N (%)	
1 Positive Test	13 (65%)
2 Positive Tests	5 (25%)
3 Positive Tests	2 (10%)
Time Since COVID-19 Diagnosis, mean (range)	21.6 months (4–37)
Medical Comorbidities, N (%)	
Hypertension	2 (10%)
Hyperlipidemia	4 (20%)
Diabetes	1 (5%)
Smoking History	1 (5%)
Anxiety	8 (40%)
Depression	6 (30%)

### 3.2 Active stand test and 6MWT

At baseline testing (N = 20), 20% of the participants experienced orthostatic tachycardia (HR increase >30 bpm), 5% experienced orthostatic hypotension (decrease by ≥ 20 mmHg in systolic blood pressure [SBP]) or ≥10 mmHg in diastolic blood pressure [DBP]); however, 95% reported at least 2 symptoms of orthostatic intolerance or dysautonomia (e.g., pre-syncope, dizziness, lightheadedness, cognitive dysfunction/“brain fog”, chest or abdominal discomfort, extremity skin color change or swelling, temperature change). In participants who presented for post-intervention testing (N = 16), 25% of participants experienced a HR increase >30 bpm, no participants had a significant change in BP and all participants reported 2 or more symptoms of orthostatic intolerance. Overall, there were no significant changes from pre to post intervention in the active stand testing, as presented in Supplemental Table 1.

The functional capacity assessment 6MWT demonstrated an increase in mean distance from 358.8 to 377.1 m from pre-to post-intervention, though this difference was not statistically significant (*p* = 0.29; Supplemental Fig. 1). Seven participants (44%) had no clinically meaningful change (≥30 m) in distance walked, while 5 (31%) and 4 (25%) had a clinically meaningful increase and decrease, respectively.

### 3.3 Self-reported physical and psychological questionnaires

Mean scores for psychological and behavioral outcomes are shown in Table 2. The mindfulness intervention led to a significant reduction in insomnia severity and a trend toward significant reduction in anxiety post-intervention. Trends toward improvements in mean scores for perceived stress, depression, well-being, COVID-19-related distress, and autonomic symptoms were seen; however, these findings were not statistically significant. No differences for resilience, fatigue or QOL were found. Additionally, there were no significant findings in the 4-week post-intervention follow-up questionnaire data; notably, the post-intervention improvements in insomnia severity and anxiety generally reverted toward their pre-intervention values (Supplemental Fig. 2).

**Table 2:** Symptom assessment scores.

Symptom Domain	Pre-Intervention (N = 17)	Post-Intervention (N = 17)	*p value*
	Mean	Mean	
Dysautonomia (COMPASS-31)	47.88	46.25	0.48
Orthostatic Intolerance	6.53	6.18	0.39
Fatigue (FSI)	78.65	80.29	0.80
Perceived Stress (PSS)	23.00	21.35	0.29
Anxiety (GAD-7)	10.18	8.10	0.059
Resilience (CD-RISC)	23.29	22.88	0.72
Depression (PHQ-8)	11.59	10.76	0.40
Event-Related Distress (IES-R)	26.53	24.65	0.57
Quality of Life (SF-20)	43.35	43.35	1.00
Insomnia (ISI)	16.65	13.65	0.001
Well-Being (MHC-SF)	36.65	37.94	0.31

### 3.4 Pro-inflammatory gene expression

In the primary analysis examining the pro-inflammatory CTRA gene expression profile, results showed a statistically significant decline from pre-to post-intervention (p = 0.004; Fig. 2A). Similar results emerged when analyses additionally controlled for any changes in leukocyte subset abundance over time (using mRNA markers of CD4^+^ and CD8^+^ T cells, B cells, NK cells, and monocytes), demographic characteristics (e.g., age, ethnicity, education, marital status, employment status, income, etc.), pre-existing conditions (depression, anxiety, PTSD, smoking, HLD, DM, HTN, mindfulness practice, etc.) or behavioral and psychometric measures that were provided. In analyses examining factors that might modify the magnitude of change over time in CTRA gene expression, results showed that declines in CTRA gene expression were most pronounced for people who reported the most COVID-19 positive events from diagnosis to study entry (omnibus p = 0.025), with the most significant reductions among those with 3 COVID-19 positive events (p = 0.010), followed by 2 COVID-19 positive events (p = 0.036) and 1 COVID-19 positive event (p = 0.050). Declines in CTRA gene expression did not vary significantly as a function of recent illness or history of COVID-19 hospitalization.

**Fig. 2:**
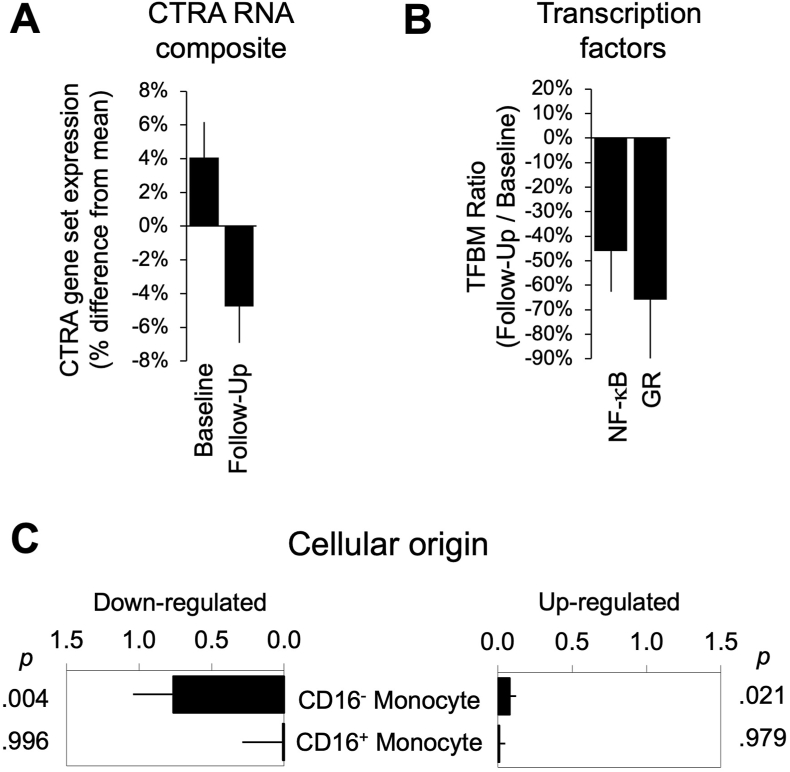
Change in Inflammatory Gene Expression. Peripheral blood samples collected at baseline and follow-up were subject to genome-wide transcriptional profiling, with analyses testing for statistically significant change in: **(A)** Average expression of 48 pre-specified gene transcripts measuring the Conserved Transcriptional Response to Adversity (CTRA), **(B)** Promoter-based bioinformatic analysis of pro-inflammatory (NF-κB) and stress-related (glucocorticoid receptor; GR) transcription factor activity for all genes showing >1.5-fold change in expression over time, and **(C)** Cellular origin in classical (CD16^−^) monocytes vs. non-classical (CD16^+^) monocytes for all genes showing >1.5-fold change in expression over time. Abbreviations: TFBM, transcription-factor binding motif.

Validating the initial analysis of the pre-specified CTRA gene contrast, converging results also emerged from secondary bioinformatic analyses that took as input all genes showing >1.5-fold change over time (i.e., unbiased genome-wide analysis; 3763 up-regulated and 72 down-regulated), and tested whether these empirical changes in gene expression might be 1) attributable to reduced inflammatory gene regulation (NF-κB) or decreased activity of the stress-related glucocorticoid receptor (GR); or 2) derive disproportionately from classical monocytes. Results implicated classical monocytes as the primary cellular mediator of the transcriptional down-regulation from pre-to post-intervention (TOA cell diagnosticity score: mean = 0.76 ± SE 0.28, p = 0.004; Fig. 2B), and they indicated significant reductions in NF-κB activity (mean 0.54-fold ± SE 0.16 ratio of NF-κB response elements in promoter sequences of up-vs down-regulated genes, p = 0.016; Fig. 2C) and GR activity (0.34 ± 0.17, p = 0.007).

### 3.5 Feasibility assessment

Among the 20 women who received the mindfulness intervention (defined as attending 2 or more group classes), the mean number of classes attended was 4.75 of the 6 group classes. Pre-recorded, expert-facilitated make-up classes were offered to ensure content was not missed. Half the participants utilized a least one make-up class session and 3 of the 20 (15%) used 2–4 make up classes.

In response to the three mindfulness experience survey statements that participants completed at the end of the study period, the majority agreed that the experience was helpful (88.24%), liked the virtual group mindfulness class format (82.35%) and were likely to continue the practice of mindfulness (76.47 %).

The focused patient interviews highlighted the benefits of the mindfulness program intervention and practice. Collectively participants reported that mindfulness practice calmed the mind and body, improved sleep quality, lessened symptoms of anxiety, fear, and panic, fostered self-awareness, health perspective, life balance, and improved coping and emotional regulation. All participants identified value from the group support aspect of the intervention. The participants conveyed their lived experience and uniquely shared understanding of the impact of the debilitating symptom profiles of Long COVID, and the group provided emotional and psychological support. Difficulties reported related to active symptoms interfering with participation, and poor concentration and attention for the 2-h class length. Suggested improvements included use of practice prompts for daily self-practice, and greater practice options in frequency and duration of the class program. These results are presented in Supplemental Table 2.

## 4 Discussion

This exploratory pilot study's aim was to assess the feasibility, acceptability, and preliminary efficacy of an evidence-based mindfulness intervention program on the physical symptoms, psychological well-being, and quality of life for women burdened with Long COVID-related dysautonomia symptoms. In physical testing, no significant changes were observed for the active stand test or 6MWT from pre to post intervention. Symptom questionnaires revealed a significant reduction in insomnia severity post-intervention, but this benefit was lost at the 4-week follow-up assessment. Trends toward improvements in mean scores were observed for anxiety, perceived stress, depression, well-being, event related distress and autonomic symptoms, but these did not reach statistical significance. Pro-inflammatory CTRA gene expression decreased significantly from pre-to post-intervention.

Dysautonomia is a complex diagnosis, requiring specialized testing procedures to implicate abnormal autonomic nervous system function as a cause of symptoms, which were not performed in the present study. Nonetheless, the symptom profile captured by the COMPASS-31 scale, commonly used to assess autonomic symptoms, demonstrated moderate-to-severe (COMPASS-31 > 20) dysautonomia symptoms reported by all of our study participants (Larsen et al., 2022), with 40% of the participants scoring 50 or greater at baseline. While changes in scores were seen post-intervention (30% vs 40% with COMPASS-31 ≥ 50), scores remained high, speaking to the high symptom burden seen in this study cohort.

All the women in our study exhibited substantial symptoms of orthostatic intolerance during the active stand test, with no change identified after the six-week intervention. This result is consistent with a recent meta-analysis examining the impact of MBI on objective physiological measures of autonomic function for individuals with chronic disease, which showed a limited effect at best (Churchill et al., 2024). Additionally, our study participants demonstrated considerable functional capacity limitation during the 6MWT, with results well below normal measures of 400–700 m. While a clinically meaningful change (≥30 m) was not observed on the whole, there was a trend toward an increase in mean distance from 358.8 m at baseline to 377.1 m post-intervention. Congruently, Zou et al. examined the effects of mindful exercises on rehabilitative outcomes such as sensorimotor function and gait speed (Zou et al., 2018), finding that a mindful exercise intervention was associated with improved sensorimotor function.

Sleep disturbance and insomnia are prevalent in Long COVID symptom profiles (Alkodaymi et al., 2022). In our sample, 35% reported a moderate level of insomnia and 30% severe insomnia prior to the intervention. Although a small sample size, our study demonstrated a significant reduction in insomnia severity (ISI score 16.6 vs. 13.6; *p* = 0.001) from pre to post intervention. This result is consistent with previous studies which found mindfulness meditation interventions significantly improved sleep quality in a broad population (Rusch et al., 2019). There is strong evidence that sleep is a key driver of inflammatory and antiviral immunity (Irwin, 2019) and thus highly relevant in the context of COVID-19. For women with Long COVID dysautonomia, mindfulness practice may be useful for improving insomnia severity with potential benefit to other associated symptoms. Notably, however, a rebound of insomnia severity was observed at the 4-week follow-up assessment (Supplemental Fig. 2). This lack of sustained improvement suggests that modifications to the mindfulness intervention, such as a longer duration or other strategies to promote continued mindfulness practice, are likely necessary to achieve lasting benefit in this domain.

More recent evidence has pointed to the importance of supporting the psychological health of Long COVID patients. The prevalence of mental health conditions in Long COVID has been reported for anxiety (23%–48%) and depression (17%–48%) (Badenoch et al., 2022; Premraj et al., 2022; Rass et al., 2022). In our sample, 60% of participants reported moderate to severe levels of symptoms of anxiety and depression prior to the intervention. Our study found trends toward improvements in scores for anxiety, perceived stress, depression, COVID-19 event-related distress symptoms and well-being, but these changes were not statistically significant. While positive trends in mean scores were observed, the small sample size of our pilot study limited the ability to detect significant differences in these domains. Our findings are consistent with the results of prior studies demonstrating the efficacy of MBIs in reduction of stress, anxiety, and depressive symptoms (Blanck et al., 2018; Spijkerman et al., 2016). Our results signal the potential benefit of mindfulness as an adjunct treatment to support psychological health for women with Long COVID dysautonomia, though additional study is needed.

Debilitating Long COVID symptom profiles involve multiple biological systems, with unpredictable severity and time course that can lead to a prolonged stress response state. Links between chronic stress and the negative impact on mental and physical health are well known (Dahlgaard et al., 2019). Biochemical systems responses for adaptation involve activation of the sympathetic adrenal medullary system, the immune system and the hypothalamic-pituitary-adrenal (HPA) axis (Allen et al., 2014). Examining physiologic markers, stress induced changes of immunological processes, variations in inflammatory and antiviral responses and gene expression can aid in targeting effective interventions (Chovatiya and Medzhitov, 2014).

In the present study, transcriptomic analyses of circulating blood cells revealed a significant decrease in the pro-inflammatory CTRA gene expression profile from pre-to post-intervention. This finding is consistent with a favorable effect of the mindfulness intervention on inflammatory biology. Similar results emerged when analyses controlled for any changes in leukocyte subset abundance over time. Interestingly, the declines in CTRA gene expression were most pronounced for people who reported the most COVID-19 positive events from diagnosis to study entry, with the most significant reductions among those with 3 SARS CoV-2 infections, followed by 2 infections, and 1 infection. Declines in CTRA gene expression did not vary significantly as a function of recent illness, COVID-19 hospitalization, demographic characteristics, or general medical history. Bioinformatic results from alternative analyses of genome-wide transcriptional changes produced convergent results, with results implicating classical monocytes as the key cellular mediator of transcriptional changes from pre-to post-intervention, and reductions in NF-κB (inflammatory) and GR (HPA axis stress response) as key transcription control pathways driving observed changes. These results are consistent with the known cellular and gene regulatory pathways through which stress and sympathetic nervous system activity regulate CTRA gene expression (Cole, 2019), and these mechanistic indications also provide cellular and molecular targets for future follow-up studies.

Notably, while there was no control group in the present study, CTRA gene expression has been relatively stable over time in previous control groups from randomized controlled (e.g., varying by < 0.05 z-scored log2 RNA abundance from baseline to follow-up) (Nelson-Coffey et al., 2017; Regan et al., 2022). By contrast, this study demonstrated a post-intervention CTRA reduction of more than twice that magnitude (−0.13 z-scored log2 RNA abundance), suggesting that the observed change was unlikely due to secular trends or random sampling error. Further, the long duration between initial COVID-19 diagnosis and the study intervention (mean 21.6 months; Table 1) may suggest that a spontaneous decrease in CTRA gene expression after acute infection is less likely to play a role. Nonetheless, given the lack of a control group for comparison, we are unable to determine the impact of the passage of time in the observed decline in CTRA gene expression.

These findings are consistent with several studies that have shown that components of CTRA - pro-inflammatory gene expression and inflammatory signaling - can change following interventions such as cognitive behavioral stress management (Antoni et al., 2012, 2016) and mindfulness meditation (Black et al., 2015; Bower et al., 2015; Creswell et al., 2012). Additionally, our findings were congruent with previous studies demonstrating significant reductions in proinflammatory gene expression and inflammatory signaling after MAPs intervention (Bower et al., 2015, 2021, 2023). Mindfulness meditation interventions have been suggested to enhance immunity and reduced inflammatory-driven pathogenesis for those with post-viral conditions such as ME/CFS and Long COVID (Porter and Jason, 2022). The findings from our pilot study suggest that an MBI may be beneficial in treatment for women Long COVID dysautonomia symptoms, balancing pro-inflammatory and anti-viral processes.

### 4.1 Limitations

Limitations of this study include a small sample, limiting power for statistically significant associations and effect sizes from the intervention to measured outcomes, including in the secondary analyses of our transcriptomic data. The small sample size also constrained the ability to perform subgroup analyses, such as whether findings were impacted by antidepressant medication use; future research should be powered to address this question. Additionally, this pilot study was non-randomized and importantly lacked a control group necessary to optimize causal inference, particularly for the decline in CTRA gene expression observed. Notably, selection bias must be recognized given a convenience sample was recruited from the center's Long COVID Program. Internal validity directs examination of potential confounding factors that may influence outcomes beyond the intervention. For example, participants identified considerable value in the intervention group format which afforded the opportunity to gain support from others who experienced similar physical and psychological challenges that may have contributed to improved outcomes. It will be important to reproduce these findings in a larger trial and determine whether the effects are generalizable to other populations. Future studies should compare a mindfulness intervention with an active control group, consider longer duration MBI (particularly given the rebound in symptom severity seen at the 4-week post-intervention follow-up in this study) or one which is part of a multimodal intervention for greater effect, and include a longer follow-up to assess sustained effects on physical, psychological, and biological outcomes.

## 5 Conclusion

This pilot study demonstrated a virtual, six-week mindfulness program may improve sleep quality in women with Long COVID dysautonomia. However, a longer intervention or other methods to promote continued mindfulness practice may be needed to achieve sustained improvement. Additionally, while no improvement in objective assessments of dysautonomia symptoms were seen, our findings indicate there may be a favorable effect of the mindfulness intervention on inflammatory and antiviral biology with a decrease in CTRA, particularly in women with a greater number of COVID-19 exposures. Our results provide support to the use of a MBI as a practical, effective, complementary approach that is feasible and acceptable. Further exploration of the efficacy of MBIs in this population is necessary with larger, controlled, multi-site studies to provide the necessary scrutiny to advance this clinical inquiry.

## CRediT authorship contribution statement

**Elizabeth Vandenbogaart:** Writing – review & editing, Writing – original draft, Visualization, Supervision, Project administration, Methodology, Investigation, Funding acquisition, Formal analysis, Data curation, Conceptualization. **Matthew Figueroa:** Writing – review & editing, Visualization, Software, Project administration, Methodology, Formal analysis, Data curation, Conceptualization. **Diana Winston:** Writing – review & editing, Resources, Methodology, Conceptualization. **Steve Cole:** Writing – review & editing, Writing – original draft, Validation, Supervision, Methodology, Investigation, Formal analysis, Data curation. **Julienne Bower:** Writing – review & editing, Visualization, Resources, Methodology, Conceptualization. **Jeffrey J. Hsu:** Writing – review & editing, Visualization, Validation, Supervision, Resources, Project administration, Methodology, Investigation, Funding acquisition, Formal analysis, Data curation, Conceptualization.

## Funding

The work was made possible by residual class settlement funds in the matter of April Krueger v. Wyeth, Inc., Case No. 03-cv-2496 (US District Court, SD of Calif.).

## Declaration of competing interest

The authors declare the following financial interests/personal relationships which may be considered as potential competing interests:Editorial Board member of Brain, Behavior, Immunity - Health - J.E.B. If there are other authors, they declare that they have no known competing financial interests or personal relationships that could have appeared to influence the work reported in this paper.

## Figures and Tables

**figs1:**
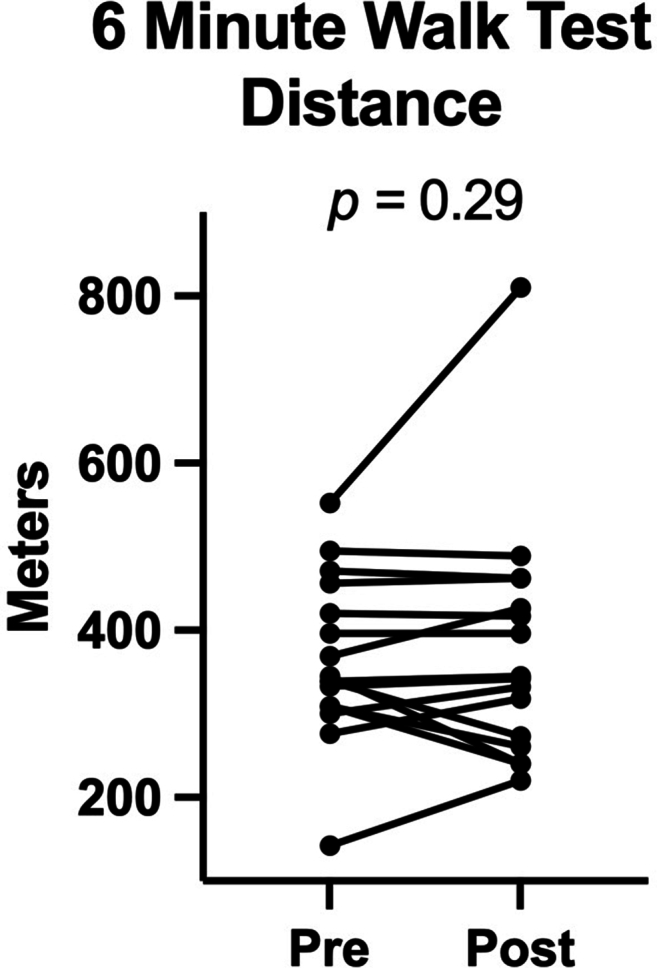


**figs2:**
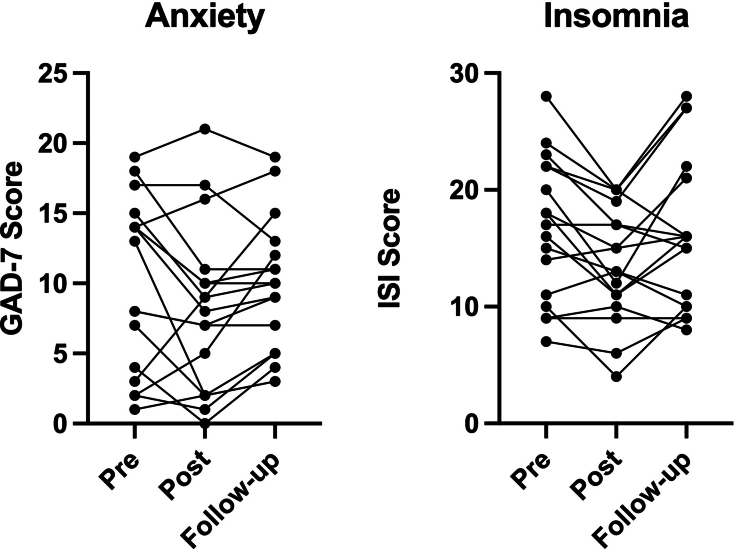


## Data Availability

Data will be made available on request.
