# Supplementary material for: Preliminary evaluation of a mindfulness intervention program in women with long COVID dysautonomia symptoms
Source: Brain Behav Immun Health. 2025 Feb 11;44:100963. doi: 10.1016/j.bbih.2025.100963 (PMC11879682; doi:10.1016/j.bbih.2025.100963)
Supplement: Multimedia component 1 [file mmc1.docx]

**Supplemental Table 1**. Active Stand Test Results.

| **Active Stand Test Findings** | **Pre-Intervention**  **(N = 20)** | **Post-Intervention**  **(N = 16)** | **P value** |
| --- | --- | --- | --- |
| Orthostatic Tachycardia  (ΔHR ≥ 30 bpm) | 20% | 25% | >0.999 |
| Orthostatic Hypotension  (Δ SBP ≥ 20 mmHg) | 5% | 0% | >0.999 |
| ≥2 Orthostatic Symptoms  (e.g., dizziness, lightheadedness, cognitive dysfunction, chest/abdominal discomfort, extremity color change, temperature change, swelling sensation) | 95% | 100% | >0.999 |

*Abbreviations:* HR, heart rate; SBP, systolic blood pressure.

**Supplemental Table 2.** Patient Experience Interview Questions, Themes, and Responses.

| **Question** | **Descriptive Themes** | **Sample of Supporting Quotes** |
| --- | --- | --- |
| In what ways did you find mindfulness helpful? | - Offers change in outlook / perspective. - Promotes self-awareness. - Promotes reflection. - Promotes balance. - Promotes relaxation. - Promotes calm. - Promotes inner peace. - Decrease states of fear, anxiety, panic. - Improves symptoms: tachycardia. - Improves Focus. - Improves ability to be present. - Improvs concentration. - Promotes emotional regulation. - Improves reactivity. - Improves coping. - Improves ability to pace activity / energy. - Improves sleep.   Program utility / format**:**   - Provided group support. - Easy virtual format. - Good tool. | “Perspective shift on our own illnesses, using mindfulness may be able to help you deal better. It’s not going to fix what’s going on, but this can give you tools to manage. It helped me engage in a group.”  “It was very calming. It also helped a lot with my sleep.”  “I found the most helpful was the element of the group. I really liked talking with other people who are going through this as well.”  “learning to calm myself during a difficult time, combat grief, I still had a couple of panic attacks, but I think it would have been more intense if I didn’t have this helpful tool.”  “Breath work was really good with addressing the racing heartbeat that I have during activity. Learning the practice, to be able to sit still and focus for longer periods.”  “It did help me a lot with the anxiety and the noises, especially at work. Because I was getting very overwhelmed with my brain.”  “I used to cry because I’d say ‘I’m never going to be myself. I’m never going to go back to the way I was. And these four weeks is like I haven’t cried. I used to cry driving. I haven’t done that.”  “I think it helped in I’m accepting this is it, this is now, and you can keep improving yourself.”  “After COVID I was like a different person, and this helped me a lot because I never thought I was going to go back to myself.”  “It’s pretty helpful. I’m always worrying. But being mindful puts you in the present time you’re in, the present space, and just being aware of everything that’s going on that you don’t really think about on a day to day because life just happens all the time. Learning breathing techniques that connect mind and body is very helpful.”  “Self-awareness in regard to pacing was extremely helpful. And my overall reactivity to my symptoms. I felt the class was super helpful. Loved the group dynamic.”  “I have never slept so good in two and a half years, and I haven’t slept that good since.”  “I had a full body sense of calmness that was really helpful. I was actually able to tell my heart rate had dropped, you know, it's beneficial to probably every human on the planet. I was more energetic and livelier. It was also helpful to be with a group of people to hear what they’re experiencing.”  “I really liked the format. I found the information easy to digest.” |
| In what ways did you find it difficult? | - Difficult to concentrate. - Attendance when symptomatic. - Fear of something new - Requires time commitment to practice. - Interruptions / distractions - Brought out difficult emotions / feelings. - Session length too long. - Materials overwhelming. | “Didn’t believe it could help until I did it.”  “Hard to stay attentive for 2 hrs.” Hard remembering to do it.”  “It brought up a lot of emotion for me.”  “Symptoms make it hard to do when you feel bad and don’t want to do one more thing.”  “Difficult to stay centered and focused. Notice you’re thinking about other things, validate it and try to recenter yourself.” |
| What do you think we can do in future to improve this mindfulness program? | Execution:   - Small groups preferred.   Program development:   - Develop self-practice prompts. - Develop app accountability / self-tracker. - Expand session options: shorter sessions, more frequent. - Multimodal, complementary inclusions | “I would prefer to be in person.”  “more useful if there was a specific mental health or support group component.”  “Two hours was really challenging.”  “When I did the makeup video it was only like an hour and a half. I wish it went on longer. I wish it was more than six weeks, but for shorter amounts of time. Just have the videos where you can do it as you go. “  “I would like the recorded sessions as well.”  “Would have liked increased frequency and length of program but short blocks of time.”  “Everyone in the group expressed that we wished it was a little longer. I think maybe that’s just because it’s so valuable that we want to keep spending time on it. I feel like the program could have been even twice as long and having multiple sessions focusing on each concept instead of a different concept each week” |
