# Supplementary material for: Preliminary evaluation of a mindfulness intervention program in women with long COVID dysautonomia symptoms
Source: Brain Behav Immun Health. 2025 Feb 11;44:100963. doi: 10.1016/j.bbih.2025.100963 (PMC11879682; doi:10.1016/j.bbih.2025.100963)
Supplement: Multimedia component 2 [file mmc2.docx]

**Supplemental Figure Legends**

**Supplemental Figure 1.** Results of 6-Minute Walk Test.

Distance walked during the test by each study participant pre- and post-intervention. Two-tailed paired *t-*test was performed.

**Supplemental Figure 2.** Anxiety and Insomnia Assessment Scores.

Individual participant scores for Anxiety (GAD-7) and Insomnia (ISI) questionnaire assessments at Pre-intervention, Post-intervention, and 4-week follow-up.
